# Vehicle Classification Using an Imbalanced Dataset Based on a Single Magnetic Sensor

**DOI:** 10.3390/s18061690

**Published:** 2018-05-24

**Authors:** Chang Xu, Yingguan Wang, Xinghe Bao, Fengrong Li

**Affiliations:** 1 Shanghai Institute of Microsystem and Information Technology, Chinese Academy of Sciences, Shanghai 200050, China; wyg@mail.sim.ac.cn (Y.W.); baoxinghe123@163.com (X.B.); fengrong.li@mail.sim.ac.cn (F.L.); 2 University of Chinese Academy of Sciences, Beijing 100049, China

**Keywords:** intelligent transport system, vehicle classification, imbalanced dataset, anisotropic magnetoresistive sensor

## Abstract

This paper aims to improve the accuracy of automatic vehicle classifiers for imbalanced datasets. Classification is made through utilizing a single anisotropic magnetoresistive sensor, with the models of vehicles involved being classified into hatchbacks, sedans, buses, and multi-purpose vehicles (MPVs). Using time domain and frequency domain features in combination with three common classification algorithms in pattern recognition, we develop a novel feature extraction method for vehicle classification. These three common classification algorithms are the k-nearest neighbor, the support vector machine, and the back-propagation neural network. Nevertheless, a problem remains with the original vehicle magnetic dataset collected being imbalanced, and may lead to inaccurate classification results. With this in mind, we propose an approach called SMOTE, which can further boost the performance of classifiers. Experimental results show that the k-nearest neighbor (KNN) classifier with the SMOTE algorithm can reach a classification accuracy of 95.46%, thus minimizing the effect of the imbalance.

## 1. Introduction

The rapid increase of vehicles contributes heavily to the air pollution in cities; thus, the optimization of urban transportation is of upmost importance. Vehicle classification plays a significant role in road maintenance, traffic flow modeling, and road safety management [1] and thus is one of the most important tasks of an intelligent transport system (ITS).

Since the 1980s, researchers have studied automatic vehicle classification systems in ITSs and concluded that the magnetic field distortion caused by vehicles can be used for vehicle detection and classification [2]. Many different sensor schemes can achieve desirable results in vehicle classification. Among them, three technologies are widely used, inductive loop detectors [3,4,5], magnetic sensor detectors [6,7,8], and vision-based detectors [9,10,11]. These three technologies have their own specific applicable scenarios. In this paper, the anisotropic magnetoresistive sensor (AMR) approach is introduced to accomplish the task of vehicle classification.

Vehicle classification based on an AMR sensor is a highly challenging but practical task. With an increasing number of vehicles on roads, the demand for automatic traffic monitoring system is growing. Therefore, a low-cost vehicle detection system, called Sensys Networks’ VDS240 [12], sprung into popularity. Using its small wireless AMR sensor nodes on lanes, the system captures the magnetic signal caused by vehicles, which can be used for vehicle detection. As a result, the study of vehicle classification based on a single magnetic sensor is crucial for automatic monitoring systems, allowing vehicle types to be introduced into other similar vehicle detection systems.

Great strides in automatic monitoring systems and ITSs are being made with the development of artificial intelligence technology [13]. In our previous work, a feature extraction and comparison method was presented for vehicle classification with a single magnetic sensor. The core lies in the smart feature extraction method and correct selection of classification algorithms. The experimental simulation results show that the proposed approach can reach 83.62% classification accuracy, which is essentially a time-domain-based method [14]. Similarly, some researchers classify vehicles by using the frequency domain methods. For example, Zhang et al. [15] has proposed one based on a frequency domain energy spectrum of a geomagnetic sensor for real-time vehicle classification, achieving an average accuracy above 90%.

There is, however, a crippling limitation. Traditional machine learning classification researches are based on the assumption that the number of samples in a dataset is relatively balanced. Unfortunately, this assumption does not hold true in traffic applications and many models are imbalanced. In Chinese cities for instance, the number of sedans is much higher than that of buses. So far, no satisfactory solution has been provided to solve this issue. Some studies have directly constructed vehicle classifiers by using a balanced dataset [8], while others avoid discussing the influence of imbalanced vehicle datasets [16].

Sections in this paper are as follows. Section 2 introduces the designed data acquisition system, which is composed of a single magnetic sensor, a camera, and a laptop. Section 3 introduces the signal preprocessing method and lists the obtained original vehicle magnetic dataset. An innovative feature extraction method is proposed in Section 4, which will be used to construct feature sets. However, the vehicle classifiers have poor performance on rare vehicle types, and the reasons are given in Section 5. Section 6 proposes an oversampling approach, which is called the SMOTE algorithm. Not only will the SMOTE algorithm solve the issue of imbalanced vehicle datasets, but it will also improve the performance of classifiers. Experimental results and discussions are presented in Section 6, which confirms that the SMOTE algorithm does indeed work. Conclusions are then given in Section 7.

## 2. Data Acquisition System Overview

When a vehicle passes close to a magnetic sensor, it will detect all of the different dipole moments of its various parts. Sufficient information could be obtained from the magnetic field distortion, including the trajectory of target metal objects and other intrinsic parameters [17]. Moreover, different types of vehicles come in different sizes and structures, leading to different magnetic field distortion. In summary, vehicle classification can be realized based on the magnetic sensor. The data gathered on vehicle types can then help to improve existing traffic monitoring systems.

**Figure 1:**
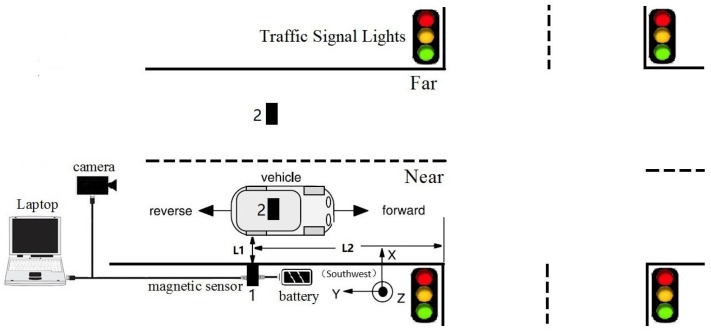
Sensor installation diagram.

As shown in Figure 1, a magnetic sensor was used to capture the magnetic signal caused by vehicles, while vehicle types were recorded by a camera. In our experiments, the HMR2300, a three-axis smart digital magnetometer, was chosen as the magnetic sensor. The HMR2300’s advantage lies in its ability to detect the strength and direction of a magnetic field and is therefore able to feed the x, y, and z components directly to a computer. Moreover, it has a range of ±2 Gauss, with a 67 μGauss resolution. In addition, the direction of the magnetic sensor installation is shown in the lower right corner of Figure 1, with its *Y*-axis pointing towards the opposite direction (southwest) parallel to the moving vehicle.

The sampling rate of the HMR2300 was set to 123 samples/s, which is close to the rate of the magnetic sensor in VDS240 (128 samples/s). We used this setup to collect magnetic data on real life traffic, in order to obtain information on behaviors of real life traffic flow. Please note that it is a two-way four-lane intersection, while the data acquisition system is deployed in a one-way two-lane intersection, where the speed is limited to 80 km/h. In addition, the distance between the sensor and the crossroads (L2 in Figure 1) was limited to 50–100 m. This distance is used to install the magnetic sensor, where the speed of the detected car is low, yet it rarely stops. According to China’s traffic laws, the vehicle must slow down at least 100 m from the intersection. As a result, vehicle speeds were already reduced before passing the sensor, and the data indicates that most vehicle speeds were less than 20 km/h. In summary, there are some advantages to setting the length of L2 as 50–100 m. For example, if the speed of the detected vehicle is lower, then we can obtain more sample points at the same sampling rate. Additionally, due to the presence of the camera at the crossroads, driving behavior will be more civilized (no bad driving behavior such as lane change or overtaking), which led to more robust magnetic data collection.

**Figure 2:**
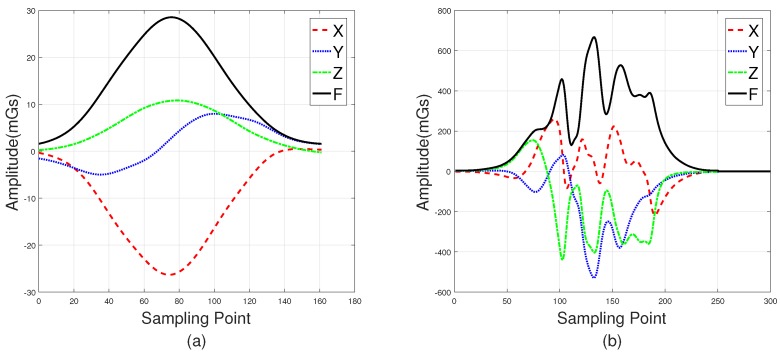
Waveforms of a sedan collected roadside are shown in (**a**). Waveforms collected in the middle of the lanes are shown in (**b**).

The roadside (Position 1 in Figure 1) and the middle of lanes (Position 2 in Figure 1) are two common positions in which sensors can be installed. Figure 2a shows three-axis magnetic signals of X, Y, and Z and the F signal (the magnetic field intensity of the measured signal) of a sedan collected roadside, with Figure 2b showing data collected through a sensor in the middles of lanes. Intuitively, the magnetic disturbance signals collected by these two deployment methods are different, and the magnetic field intensity captured by the middle-of-lane installation scheme is about 24 times larger than the one on roadside. Both of these two sensor installation schemes are able to realize vehicle classification, and each have their own applicable scenarios. However, from the view of deployment, the roadside method is simpler, while the one in the middle of lanes is more time-consuming and may even disrupt traffic. Thus, the roadside installation was chosen for capturing magnetic signals in our experiments.

Note that, in Figure 2, the two waveforms contain 120 samples and 200 samples, respectively. This is because L2—the distance between the detected car and the sensor—of these two deployment methods are different. In addition, the distance between the detected car and the magnetic sensor (L1 in Figure 1) changes with the mounted height, which directly leads to different magnetic field distortion. In other words, the height suitable for different types of vehicle are not the same, which increases the difficulty of vehicle classification. In this study, the magnetic sensor was installed close to the ground, allowing it to detect all vehicle classes. This may not be the most optimal solution, but it is the simplest, and the classification results validate this. Considering that the height parameter is a key factor, we plan to find out the optimal value for different classes of vehicles in future studies.

## 3. Vehicle Classification Magnetic Database

### 3.1. Signal Preprocessing

Magnetic signal data collected through either method in the previous section contain noise, negatively impacting our aim to classify vehicles. Therefore, in order to obtain accurate magnetic field disturbance caused by vehicles, the raw signals need to be processed by signal preprocessing steps: signal filtering, signal background removing, and signal segmentation [14].

Simulation results have shown that the magnetic field distortion caused by vehicles is of low-frequency, while the noise signals in the environment are usually high-frequency [18]. Hence, a low-pass filter is recommended to remove the high-frequency noise. The low-pass filter bandwidth is set to half of the sampling frequency, which is 62 Hz. The intensity and direction of Earth’s magnetic field also vary with location, so the next step is to remove the background value of the magnetic field. In this study, the background value is defined as the one detected when there are no vehicles passing by. Finally, signal segmentation can be achieved by setting appropriate thresholds, with the goal of extracting the useful parts of the magnetic signal caused by vehicles.

### 3.2. Original Vehicle Classification Magnetic Database

There are several vehicle classification standards in the world, and the one used in this paper is the FHWA13-Category Scheme, which is recommended by the Federal Highway Administration (FHWA) in the United States. However, there is a big difference among the number of these types under different traffic scenarios. In our experiments, the magnetic data was captured in urban traffic, which is mainly composed of hatchbacks, sedans, multi-purpose vehicles (MPVs), and buses.

As shown in Table 1, the obtained magnetic database covers a wide range of vehicle types, including motorcycles, *hatchbacks*, *sedans*, *MPVs*, *buses*, and trucks. Furthermore, the *Original Set* is randomly divided into the *Training Set* and the *Testing Set*, and the size of the former is set to be about 3 times larger than the latter. Thus, motorcycles and trucks are not discussed in this paper, due to the lack of data. There are eight types of trucks in the FHWA13-Category Scheme, and their length and structures are different. Furthermore, the cargo on the trucks may bring negative impacts on the classification results, and the cargo’s information is unknown. For achieving vehicle classification for these trucks, we not only need to collect sufficient data but also investigate the impacts of cargo. In summary, the most common types are discussed in this paper, and the classification for motorcycles and trucks will be investigated in future research.

Note that the proposed system cannot handle static vehicles, as the AMR sensor can only detect a moving car. In the process of data acquisition, fewer than 10 profiles were caused by stop-and-go cars, which have been removed from the original database.

## 4. Feature Extraction

Based on the magnetic database above, this section will introduce an innovative feature extraction method, which is essentially the fusion of time-domain and frequency-domain methods. The raw magnetic signal consists of the *X*-, *Y*-, and *Z*-axis signals, and the magnetic field intensity of measured signals (notated as *F*) can be easily calculated using Equation (1). There are four signal waveforms in total, whose features will be extracted in this section.

(1) $$F=\sqrt{(X^{2}+Y^{2}+Z^{2}).}$$

**Figure 3:**
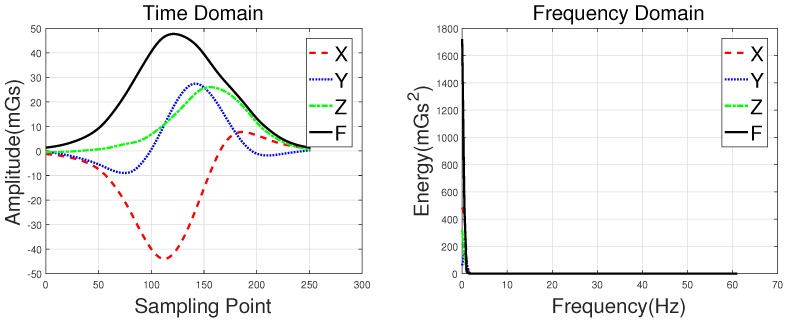
Waveforms of a hatchback and their frequency spectrums.

**Figure 4:**
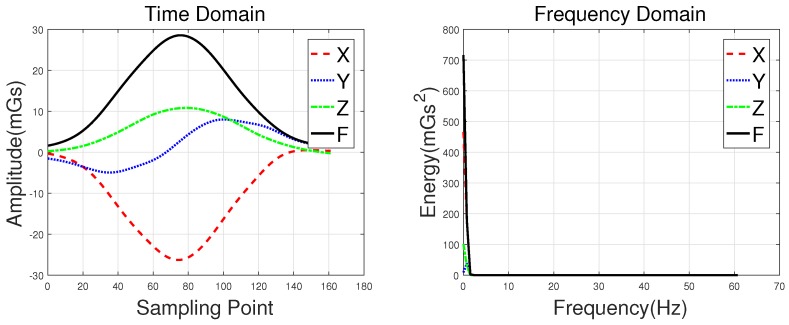
Waveforms of a sedan and their frequency spectrums.

**Figure 5:**
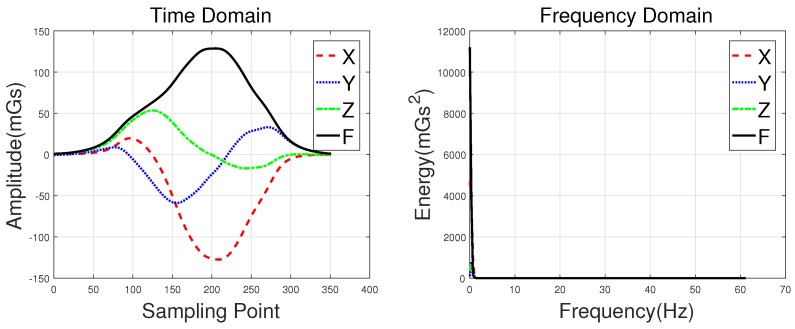
Waveforms of a bus and their frequency spectrums.

**Figure 6:**
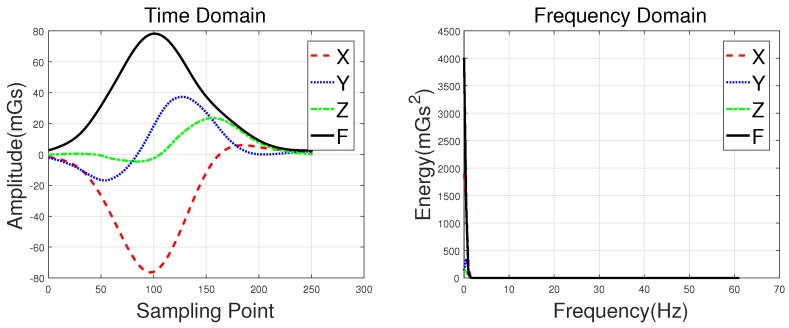
Waveforms of an MPV and their frequency spectrums.

Waveforms of a hatchback, a sedan, a bus, and an MPV along with their frequency spectrums are shown in Figure 3, Figure 4, Figure 5 and Figure 6, with the upper subgraphs consisting of the *X*-, *Y*-, and *Z*-axis components and the curve of the *F* signal in the time domain. Figures on the lower subgraphs are the corresponding signal waveforms in the frequency domain. There is significant difference among these waveforms, which is mainly caused by the vehicles detected belonging to different classes. In this paper, the detected vehicles are classified into hatchbacks, sedans, buses, and MPVs. For the magnetic sensor, the difference between these types is mainly reflected in length, structure, and the distribution of ferromagnetic materials of the vehicles. Their frequency spectrums can be easily obtained by using the fast Fourier transform (FFT) method. In this study, *X*, *Y*, *Z* represent the feature sets extracted from the *X*-, *Y*-, and *Z*-axis triaxial magnetic field components, and F represents the feature sets extracted from the magnetic field strength. For brevity, the feature set constructed based on the time domain method is notated as *Time*, and the one obtained by the frequency domain method is notated as *Frequency*.

First, let us introduce the *Time* feature set. Its definition is shown in Equation (2).

(2) Time={PMax/L,PMin/L,PP,Me,Std,NoExt,ASign,ZCNo,E,AvgE,RtoE,L}.

*PMax* and *PMin* are defined as the position of the maximum and the position of the minimum, and *L* is the sampling points of the detected signal. *PP*, *Me*, and *Std* are peak-to-peak value, mean value, and standard deviation value of the detected signal, respectively. *NoExt*, *ASign*, and *ZCNo* are the number of extremes, the sign of the first extreme, and the number of zero-crossing. *E* is defined as the energy of the detected signal, and *AvgE* is the ratio of *E* to *L*. Lastly, *RtoE* is defined as the ratio of the energy of the *X*-, *Y*-, and *Z*-axis signals to the energy of the *F* signal (e.g., *RtoE_X* is the ratio of *E_X* to *E_F*). According to Equation (1), it is clear that the value of the *F* signal is non-negative, whose DC component has been removed. As a result, the *PMin*, *ASign*, and *ZCNo* of the *F* signal are not included in the *Time* set. The *RtoE* feature is also not adapted for the *F* signal, because its value is always equal to 1.

An FFT is an algorithm that samples a signal over a period of time (or space) and divides it into its frequency components. In this study, we used the *fft* function in MATLAB to achieve the signal transformation process, which is shown in Equation (3).

(3) W=fft(T).

*T* represents the obtained magnetic signals ( the *X*-, *Y*-, and *Z*-axis signals and the *F* signal), and *W* is the discrete Fourier transform (DFT) of vector *T*.

However, *W* is a sequence of complex numbers, which is not suitable for further processing. It needs to undergo conversions in order to be used. In this study, the energy spectrum is chosen for extraction features in the frequency domain, which is an often-used method in the signal processing field. As shown in Equation (4), the energy is the square of the magnitude of the frequency signal.

(4) $$WE=AMP^{2}.$$

The signal energy is notated as *WE*, and *AMP* is the magnitude of the frequency signal, which is calculated in Equation (5).

(5) $$\begin{array}{c} AMP=2|W|/L\\ AMP(1)=AMP(1)/2. \end{array}$$

L is the number of sampling points, and *AMP*(1) is the DC component of the frequency signal.

Based on the energy spectrum curves, we can obtain 2∼9 non-zero samples of the frequency spectrum, which limits a reliable estimation of the features extracted in the frequency domain. In other words, feature extraction based on 2∼9 non-zero samples does not make sense. A simple solution is directly using these non-zero samples to construct the Frequency feature set, which is shown below.

(6) Frequency=WE(k).

WE(k) is the first k points of the WE (defined in Equation (4)), whose value is 9 in this paper.

In conclusion, the *Feature* set is defined as Equation (7), which is the fusion of the *Time* set and the *Frequency* set.

(7) Feature={Time,Frequency}.

In order to simplify the model, this study is under a strong assumption: When the detected vehicle passes by the magnetic sensor, its speed is uniform. This assumption is inaccurate in most scenarios, but we can assume it is approximately correct in this study. The reasons are as follows. First, the detected vehicle’s speed is low, and the driving behavior is civilized, both of which are thanks to the smart sensor’s installation position. Secondly, most features in the Feature set are not easily affected by the speed, and those that are have been optimized by speed normalization. For example, both PMax and PMin are easily affected by speed, so the ratio between them and L (the sampling points of the detected signal) have been used in the Time set instead. With the sampling ratio at 123 samples/s (notated as Fre), the speed (notated as V) can be easily calculated using Equation (8):

(8) V=(Fre∗Len)/L.

Len is the length of the vehicle.

From the above equation, we can reach a conclusion that V is inversely proportional to L. Furthermore, Len values of a single vehicle model are relatively close. Lastly, features easily affected by speed account for a smaller proportion in the Feature set, which leads to a smaller influence on the classification results. Thus, the influence of speed has a minimal effect on the feature extraction method proposed in this study, as proven mathematically. In other words, as long as the sampling data is sufficient, the feature extraction method proposed in this paper can work well at a higher speed.

**Figure 7:**
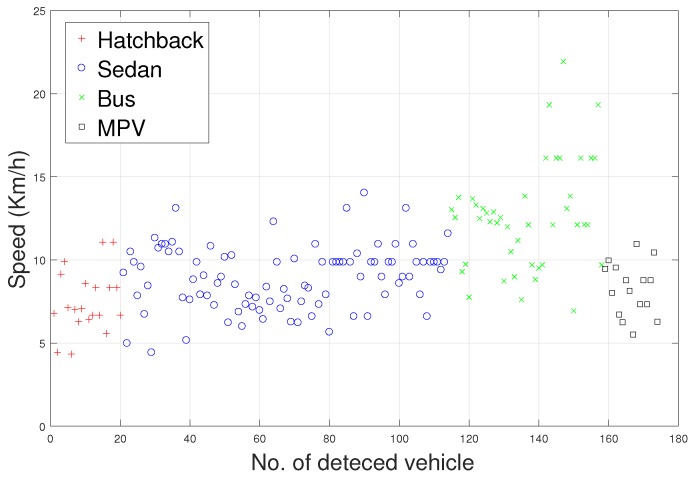
The speeds of detected vehicle cars.

The speed of the detected vehicle directly determines the number of sampling points that can be obtained, and has a significant effect on the magnetic disturbance waveform. As mentioned earlier, the sampling rate of the HMR2300 was set to 123 samples/s. In this paper, we assume that the average lengths of the hatchbacks, sedans, buses, and MPVs are 3.8, 4.5, 11, and 5 m, respectively. Thus, according to Equation (8), the approximate speeds of the detected vehicles can be calculated. As shown in Figure 7, the speed of the vehicles collected in the original database is approximately 4–23 km/h. In the obtained database, the number of sampling points (time domain) is in the range of 142–702, and these data have the ability to distinguish the models discussed in this paper.

## 5. Vehicle Classification

### 5.1. Classification Algorithms

In this study, the aim of vehicle classification is to classify the detected vehicles into the following types: hatchbacks, sedans, buses, and MPVs. Based on the obtained *Feature* set, vehicle classification can be accomplished using the following three common classification algorithms: the k-nearest neighbor (KNN), the support vector machine (SVM), and the back-propagation neural network (BPNN).

To begin with, the KNN [19] is a simple yet effective method in pattern recognition. Its input consists of training examples in the feature space closest to K and has a class membership output. In essence, an object is classified by a majority vote of its neighbors, with the object being assigned to the class most common among its k-nearest neighbors (k is a positive integer, typically small).

An SVM [20] constructs a hyperplane or set of hyperplanes in a high- or infinite-dimensional space, which can be used for classification. In fact, the SVM is a maximum margin classifier. In other words, it supposes that the best separation is achieved by the hyperplane that has the largest distance to the closest points in the training set, which are termed as support vectors.

For the final step, the BPNN [21], one of the most widely used neural networks, is used. It calculates a gradient that is needed in the calculation of the weights to be used in the network. Then, the gradient is fed to the optimization method, which in turn uses it to update the weights in an attempt to minimize the loss function.

### 5.2. Classification Results Based on *Feature* Set

In this study, the automatic vehicle classifiers are constructed based on the training set, and their performance is validated on the testing set. From Table 1, it is clear that the original database is imbalanced (that is, the numbers of observations in different classes vary greatly). For example, there are magnetic data of 94 sedans and 16 MPVs in the *Original* Set, with the number of the former about 5 times greater than that of the latter. However, accuracy is not a reliable metric for the real performance of a classifier, as it will yield misleading results if the dataset is imbalanced. As a result, the F1-Measure method is introduced in this section, and the vehicle classification results are shown in Table 2.

By comparing the accuracies in Table 2, we find that the BPNN algorithm obtains the highest classification accuracy of 81.82%, a performance that is better than others. Intuitively, this result is acceptable, but we wish to obtain a better classification result.

### 5.3. Negative Impact Caused by the *Imbalanced Dataset*

It is known to us that the F1 is the harmonic average of the precision and recall, and it reaches its best value at 1 (perfect precision and recall) and worst at 0 [22]. Moreover, precision is the number of correct positive results divided by the number of all positive results, and recall is the number of correct positive results divided by the number of positive results that should have been returned. By introducing the F1-Measure method, we can obtain the classification results of each vehicle type, which is good for finding a better classification scheme.

To give a clear idea of the F1-Measure method, a table of confusion (sometimes also called a confusion matrix) is given in Table 3. These results are the ones obtained using the BPNN method in Table 2. As mentioned above, the performance of vehicle classifiers is tested based on the *Testing* Set, which consists of 5 hatchbacks, 24 sedans, 11 buses, and 4 MPVs (shown in Table 1). Moreover, by using the BPNN method, the predicted classes are 0 hatchbacks, 31 sedans, 11 buses, and 2 MPVs. From Table 3, we can easily calculate *Precision* and *Recall*, which are shown in Equations (9) and (10).

(9) $$\begin{array}{c} Precision=NCC/NAC. \end{array}$$

*NCC* is the number of correct classification for a class, and *NAC* is the number of the corresponding actual class.

(10) $$\begin{array}{c} Recall=NCC/NAP. \end{array}$$

*NAP* is the number of the corresponding predicted class.

According to the definition of F1-Measure, the F1 value can be calculated using *Precision* and *Recall*, which is given in Equation (11).

(11) $$F1=\frac{2*Precision*Recall}{Precision+Recall}.$$

For example, for the sedan class in Table 3, its values of *NCC*, *NAC*, and *NAP* are 24, 24, and 31, respectively. The *Precision*, *Recall*, and *F1* values can thus be obtained based on Equations (9)–(11), and the results are listed in Table 2.

By comparing the *F1* values of each vehicle type in Table 2, we discover that the vehicle classifiers perform well for sedans and buses, but have a poor performance for hatchbacks and MPVs. From Table 1, it is conspicuous that the magnetic data of sedans and buses are more sufficient, and the data of hatchbacks and MPVs are insufficient. In other words, the classifiers constructed with an imbalanced dataset have a good prediction for *Majority*, and a poor performance for *Minority*. This problem is defined as *Imbalanced Database* in this study and contributes to the negative impact on the task of vehicle classification. Nevertheless, sometimes the prediction of *Minority* is crucial. For example, pavement management processes take large cars into account because they can damage the road surface [23]. To tackle this issue, an oversampling approach has been introduced to solve the problem of *Imbalanced Database*, which is called the *SMOTE* algorithm.

## 6. Solution to the Problem of *Imbalanced Database*

### 6.1. SMOTE Database

Based on the classification results from above, two major challenges must be overcome to improve the performance of vehicle classifiers. First, in the process of feature extraction for vehicle classification, “feature engineering” is inevitable. Second, the *Imbalanced Database* bias towards *Majority* vehicles results in poor performance in regard to Minority vehicles. In face of these two challenges, the former cannot be avoided, but we can give an excellent solution to the latter one.

Addressing the issue of *Imbalanced Database*, there are mainly two solutions: from the perspective of the dataset, we can reconstruct the training set. The other solution is to make the algorithm smarter. In this section, we will propose an oversampling method called SMOTE [24] that addresses the first solution listed to solve the problem of *Imbalanced Database*. According to certain rules, the SMOTE algorithm randomly generates new *Minority* sample points, which will be merged into the original training set in order to generate a new one. In other words, the performance improvement is achieved by adding a number of *Minority* samples.

The remodeling dataset can be obtained by using the SMOTE algorithm to process the *Training Set* in Table 1, while keeping the *Testing Set* constant. This is due to the vehicle classifiers being constructed based on the *Training Set*, and their performance verified in the *Testing Set*. In order to obtain a balanced dataset, the *Minority* in *Training Set* should be processed by the SMOTE algorithm. Note that buses are also processed by this algorithm, which is less than half the number of sedans. Finally, the obtained *SMOTE Database* is shown in Table 4, presenting an almost balanced dataset for the *Training Set*. In fact, the numbers of hatchbacks, buses, and MPVs have been increased by 4, 2, and 5 times, respectively.

### 6.2. SMOTE Operation

The SMOTE algorithm was first proposed by Chawla et al. [24], and is an often-used approach to the construction of classifiers from imbalanced datasets. According to the authors, the minority class is over-sampled by creating “synthetic” examples rather than by over-sampling with replacement. By operating in “feature space”, the minority class is over-sampled by taking each minority class sample and introducing synthetic examples along the line segments joining any/all of the k minority class nearest neighbors. Depending upon the amount of over-sampling required, neighbors from the k-nearest neighbors are randomly chosen. Synthetic samples are generated in the following way: take the difference between the feature vector (sample) under consideration and its nearest neighbor. Multiply this difference by a random number between 0 and 1, and add it to the feature vector under consideration. This causes the selection of a random point along the line segment between two specific features.

There are some inherent similarities in magnetic disturbance data caused by the same vehicle type, and this conjecture is the basis for vehicle classification. By designing reasonable feature extraction methods to characterize these similarities, we can obtain effective automatic vehicle classifiers. In this article, the minority classes consist of hatchbacks, buses, and MPVs, which have been processed by the SMOTE approach. Furthermore, Std and PP have been chosen to display the effects caused by the SMOTE approach.

**Figure 8:**
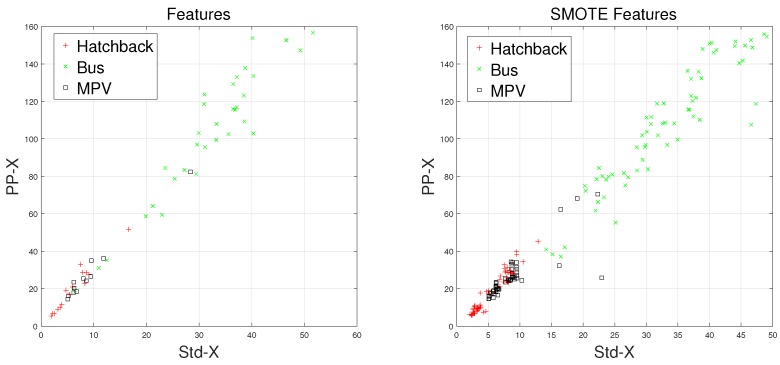
“STD-PP”-X features processed by SMOTE.

**Figure 9:**
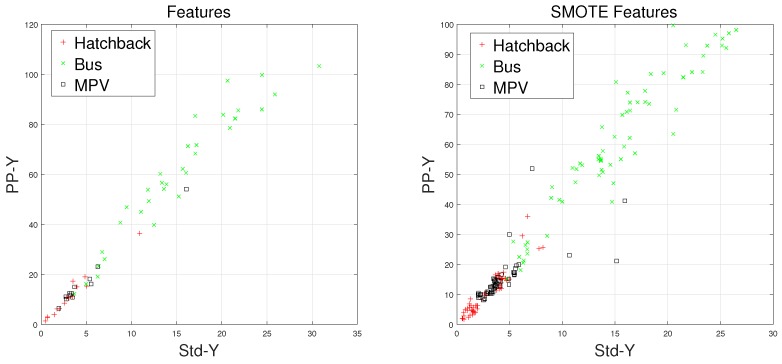
“STD-PP”-Y features processed by SMOTE.

**Figure 10:**
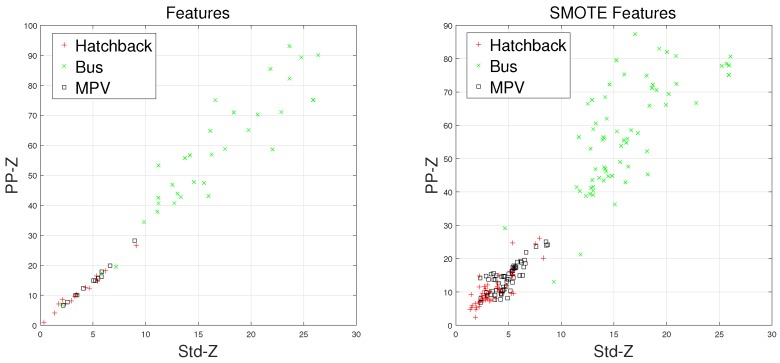
“STD-PP”-Z features processed by SMOTE.

**Figure 11:**
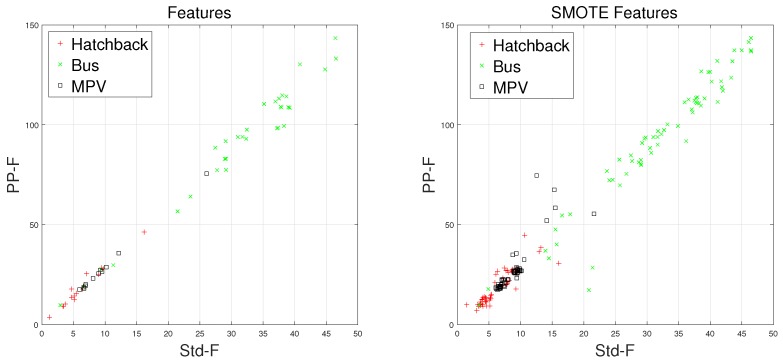
“STD-PP”-F features processed by SMOTE.

Waveforms of hatchbacks, buses, and MPVs along with their “Std-PP” features processed by the SMOTE approach are shown in Figure 8, Figure 9, Figure 10 and Figure 11, with the left subgraphs consisting of the “Std-PP” features extracted from the X-, Y-, and Z-axis components and the F signal. From these left subgraphs, we find that the values of Std and PP caused by buses are greater than those caused by hatchbacks and MPVs. Furthermore, these two features caused by the same vehicle type have a certain degree of clustering characteristics. Intuitively, this is confirmed by the fact that buses have a larger size than the other two types of vehicles and contain more ferromagnetic materials. Therefore, we have reason to believe that buses have the largest Std and PP values. After the features are processed by the SMOTE approach, some neighbors from the k-nearest neighbors are chosen. As shown in the right subgraphs (Figure 8, Figure 9, Figure 10 and Figure 11), the features in the SMOTE Database also have similar clustering characteristics. As a result, using the SMOTE algorithm to build synthetic data is reasonable, and the classification results would prove it.

### 6.3. Experiment Results Using the SMOTE Algorithm

In Section 5, the feature extraction method is discussed, but this section discusses the use of the SMOTE algorithm to generate a new *Training Set*. Based on the *SMOTE Database*, the classification results powered by SMOTE with the *Feature* set are shown in Table 5.

**Figure 12:**
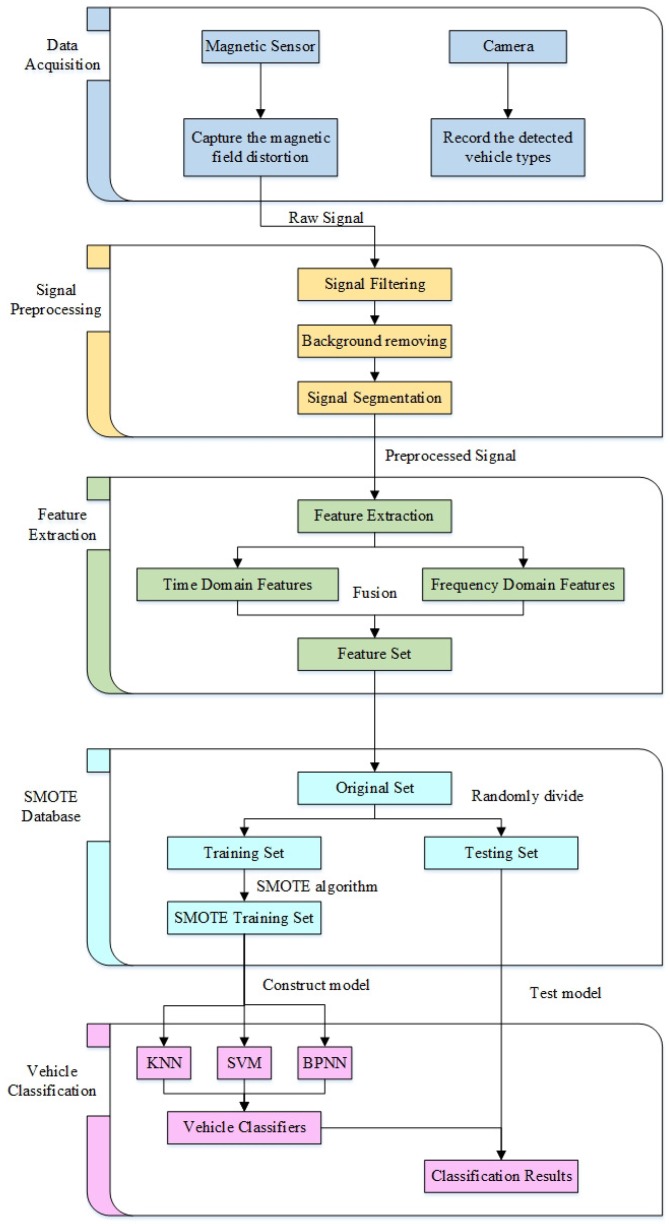
Flowchart of signal processing.

By comparing and analyzing the results in Table 2 and Table 5, we can reach the following conclusions. First, after applying the SMOTE algorithm, the classification accuracy of the KNN classifier increased from 70.46 to 95.46%, which is the highest accuracy in Table 5. Second, the performance of the SVM classifier has slightly improved, with its classification accuracy increased from 77.27 to 79.55.46%. However, the performance for Minority vehicles has not improved. This may be because the SVM classifier is a “Maximum Margin Classifier”, whose performance depends on a few “support vectors”. The BPNN classifier also saw a slight increase in classification accuracy after using the SMOTE algorithm, but instead produced an improved prediction for *Minority* vehicles. We conclude that the SMOTE method can effectively solve the problem of *Imbalanced Database* due to its role in increasing KNN accuracy and improving F1 values. The flowchart of signal processing is given in Figure 12.

## 7. Conclusions

Using discrete Fourier transform (DFT) and principal component analysis (PCA) to automatically extract features from magnetic signatures, a vehicle classification system of three categories could be made based on a single wireless magnetometer [25]. Moreover, by using the SVM algorithm, a total classification accuracy of approximately 87% was obtained. Compared to this method, our approach can not only obtain a higher classification accuracy but also cope with more types of vehicles. Furthermore, vehicle classification based on the magnetic length and average magnetic height of vehicles using a portable roadside sensor system may also achieve desirable results [7], but this sensor system requires four magnetic sensors and a GPS sensor. By contrast, our proposed method for vehicle classification only uses a single magnetic sensor while achieving comparable results.

In this study, we first introduced the data acquisition system, which has been used for constructing the original vehicle classification magnetic database. Next, a clever feature extraction method is proposed, which is the fusion of time-domain and frequency-domain methods. For improving the performance of vehicle classifiers, the F1-Measure method was introduced to contribute to the classification results of each vehicle type. By comparing the F1 values, we find that the classifiers, with an imbalanced dataset, are biased heavily toward *Majority* vehicles, leading to poor prediction for *Minority* vehicles. In order to solve this problem, the SMOTE algorithm is introduced to reconstruct the *Training Set*. Experimental results show that, after using the SMOTE algorithm, the KNN classifier with the *Feature* set reaches an excellent classification accuracy of 95.46%. In addition, the prediction of *Minority* vehicles becomes more accurate.

## Figures and Tables

**Table 1:** Original vehicle classification magnetic database. MPV: multi-purpose vehicle.

Vehicle Type	Motorcycle	Hatchback	Sedan	Bus	MPV	Truck	Total
Training Set	-	15	70	33	12	-	130
Testing Set	-	5	24	11	4	-	44
Original Set	1	20	94	44	16	3	178

**Table 2:** Vehicle classification results based on the Feature set.

Method	Index	Hatchback	Sedan	Bus	MPV
KNN	accuracy	0.7046			
	precision	0	0.7917	1	0.25
	recall	0	0.7037	1	0.3333
	F1	0	0.7451	1	0.2857
SVM	accuracy	0.7727			
	precision	0	1	0.9091	0
	recall	0	0.7059	1	0
	F1	0	0.8276	0.9524	0
BPNN	accuracy	0.8182			
	precision	0	1	1	0.25
	recall	0	0.7742	1	0.5
	F1	0	0.8727	1	0.3333

**Table 3:** Vehicle classification results based on the Feature set using the back-propagation neural network (BPNN) method.

		Predicted Class				
		Hatchback	Sedan	Bus	MPV	All
Actual Class	hatchback	0	4	0	1	5
	sedan	0	24	0	0	24
	bus	0	0	11	0	11
	MPV	0	3	0	1	4
	all	0	31	11	2	44

**Table 4:** The SMOTE Database.

Vehicle Type	Hatchback	Sedan	Bus	MPV	Total
Training Set	60	70	66	60	256
Testing Set	5	24	11	4	44
SMOTE Set	65	94	77	64	300

**Table 5:** Powered by SMOTE with Feature set.

Method	Index	Hatchback	Sedan	Bus	MPV
KNN	accuracy	0.9546			
	precision	0.8	1	1	0.75
	recall	1	0.9231	1	1
	F1	0.8889	0.96	1	0.8571
SVM	accuracy	0.7955			
	precision	0	1	1	0
	recall	0	0.7273	1	0
	F1	0	0.8421	1	0
BPNN	accuracy	0.8409			
	precision	0.2	0.9167	1	0.75
	recall	0.25	0.88	1	0.75
	F1	0.2222	0.8980	1	0.75
